# Harnessing invariant natural killer T cells to control pathological inflammation

**DOI:** 10.3389/fimmu.2022.998378

**Published:** 2022-09-15

**Authors:** Nikhila S. Bharadwaj, Jenny E. Gumperz

**Affiliations:** Department of Medical Microbiology and Immunology, University of Wisconsin School of Medicine and Public Health, Madison, WI, United States

**Keywords:** iNKT cell, CD1d, anti-inflammatory, immunotherapy, immuno-regulatory, immunomodulatory

## Abstract

Invariant natural killer T (iNKT) cells are innate T cells that are recognized for their potent immune modulatory functions. Over the last three decades, research in murine models and human observational studies have revealed that iNKT cells can act to limit inflammatory pathology in a variety of settings. Since iNKT cells are multi-functional and can promote inflammation in some contexts, understanding the mechanistic basis for their anti-inflammatory effects is critical for effectively harnessing them for clinical use. Two contrasting mechanisms have emerged to explain the anti-inflammatory activity of iNKT cells: that they drive suppressive pathways mediated by other regulatory cells, and that they may cytolytically eliminate antigen presenting cells that promote excessive inflammatory responses. How these activities are controlled and separated from their pro-inflammatory functions remains a central question. Murine iNKT cells can be divided into four functional lineages that have either pro-inflammatory (NKT1, NKT17) or anti-inflammatory (NKT2, NKT10) cytokine profiles. However, in humans these subsets are not clearly evident, and instead most iNKT cells that are CD4^+^ appear oriented towards polyfunctional (T_H0_) cytokine production, while CD4^-^ iNKT cells appear more predisposed towards cytolytic activity. Additionally, structurally distinct antigens have been shown to induce T_H1_- or T_H2_-biased responses by iNKT cells in murine models, but human iNKT cells may respond to differing levels of TCR stimulation in a way that does not neatly separate T_H1_ and T_H2_ cytokine production. We discuss the implications of these differences for translational efforts focused on the anti-inflammatory activity of iNKT cells.

## Introduction

iNKT cells are innate T lymphocytes that are present in all individuals and use a unique “semi-invariant” TCR, comprised of a canonically rearranged TCRα chain (TRAV10-TRAJ18) paired with TCRβ chains utilizing TRBV25-1 in diverse rearrangements (1–3). The TCRs of iNKT cells are specific for CD1d, a non-classical antigen presenting molecule that has minimal polymorphism at the amino acid level in human populations (4). CD1d molecules are constitutively expressed by professional APCs, including B cells, monocytes, macrophages, and DCs (5), and also by non-hematopoietic cells (particularly epithelial cells) in a variety of tissues (6). CD1d molecules are specialized for presenting lipidic antigens, which are structurally conserved molecules that are not highly mutable (7). Antigens recognized by iNKT cells derive from both self and microbial sources (8). Self-lipids recognized by iNKT cells are constitutively presented by CD1d^+^ APCs, and may also be up-regulated during inflammation or cellular stress (9). Hence, because of their status as ‘donor-unrestricted’ T cells that recognize conserved antigens and do not mediate alloreactivity, iNKT cells are ideal candidates for allogeneic cellular immunotherapies. Due to their self-lipid recognition iNKT cells can be used for adoptive cellular immunotherapies without added antigens. Alternatively, they can be specifically activated by synthetic mimetics of their lipid antigens.

Extensive studies have demonstrated remarkable potency of iNKT cells in limiting T_H1_-driven pathology in multiple settings, including autoimmune diseases, inflammation associated with obesity, and graft versus host disease (GVHD) [reviewed in (10–12)]. However, a central conundrum about iNKT cells is that they can also potently *promote* T_H1_ responses. Their T_H1_-promoting functions have been associated with enhanced defense against infections and cancer (reviewed in (13, 14)), but also appear to play pathological roles in certain contexts, including atherosclerosis, sickle cell disease, and endotoxic shock (reviewed in (15–17)). Thus, in order to successfully exploit the potential of iNKT cells to treat inflammatory disease, it may be important to selectively engage their anti-inflammatory pathways.

## How are the anti-inflammatory effects of iNKT cells mediated?

**Figure 1:**
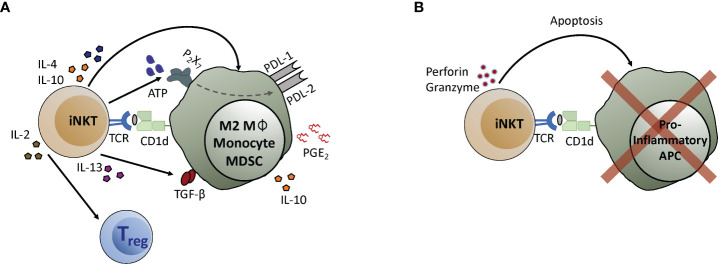
iNKT cell anti-inflammatory mechanisms. **(A)** iNKT cells interact with myeloid cell types to initiate the activation of regulatory pathways. Recognition of antigens presented by CD1d molecules expressed by myeloid cells induces iNKT cells to produce cytokines like IL-4, IL-10, or IL-13, that in turn act on the APCs. IL-4 and IL-10 promote macrophage differentiation into an M2 phenotype. IL-13 promotes monocyte differentiation into APCs that express suppressive cytokines such as IL-10 and TGF-β. Secretion of ATP by iNKT cells leads to upregulation of the checkpoint inhibitors PD-L1 and PD-L2, and iNKT interaction with monocytes induces secretion of PGE_2_ by mechanisms that have not yet been determined. Additionally, IL-2 produced by iNKT cells helps to drive the expansion of T_regs_. **(B)** iNKT cells can lyse pro-inflammatory APCs, leading to reduced T cell activation. In this case, recognition of antigens presented by CD1d molecules activates iNKT cells to release cytolytic granules that induce apoptosis of pro-inflammatory APCs.

Two distinct mechanistic processes have been identified that may explain how iNKT cells limit T_H1_-driven inflammation. The first is a regulatory axis characterized by iNKT cell production of T_H2_ (IL-4, IL-13) or regulatory (IL-10, TGFβ) cytokines, and by activation of anti-inflammatory cells including M2-polarized macrophages, myeloid-derived suppressor cells (MDSCs), and T_regs_ (**Figure 1A**). The second is a cytolytic pathway involving iNKT-mediated killing of inflammatory antigen presenting cells (APCs) that activate T_H1_ effectors (**Figure 1B**).

### iNKT regulatory axis

Studies investigating insulitis in non-obese diabetic (NOD) mice were amongst the first to elucidate the regulatory activity of iNKT cells, with early work revealing a critical link to IL-4 and IL-10 production (18–21), and further analysis showing that they promote the differentiation of tolerogenic APCs that limit the activation of autoreactive T cells (22–25). A similar axis has been observed in murine models of diet-induced obesity, where adipose-resident iNKT cells play a powerful role in glucose tolerance by promoting macrophage polarization into a non-inflammatory M2 phenotype through secretion of IL-4 and IL-10 (26, 27), and by transactivating regulatory T cells *via* secretion of IL-2 (28). iNKT cells also contribute to the resolution phase of sterile inflammation in the liver by promoting monocyte transition into an anti-inflammatory phenotype through secretion of IL-4 (29, 30). In murine models of allogeneic hematopoietic transplantation, iNKT cells protect against GVHD through IL-4 dependent mechanisms (31–33), and by promoting the regulatory functions of myeloid-derived suppressor cells (MDSCs) while driving T_reg_ expansion *via* secretion of IL-2 (34–36).

Analyses of human iNKT cells have suggested that they may participate in similar regulatory processes. IL-10 producing iNKT cells recently identified in the intestinal lamina propria of Crohn’s Disease patients showed suppressive activity towards pathogenic CD4^+^ T cells, and the frequency of IL-10 producing iNKT cells in colon tissue of these patients correlated inversely with T_H1_ and T_H17_ cell frequency, and was associated with reduced disease severity, higher *TGFB* gene expression, and lower levels of inflammatory proteins (37). Moreover, co-culture of human T_regs_ with iNKT cells led to increased T_reg_ FOXP3 expression, enhanced IL-10 secretion, and more profound inhibition of conventional T cell proliferation (38).

Human iNKT cells can also mediate potent suppression of T cell IFN-γ production by modulating the functions of monocytic cells. Our research group showed that GM-CSF and IL-13 secretion by human iNKT cells induced monocytes to differentiate into tolerogenic APCs that produced high levels of IL-10, expressed the checkpoint inhibitors PDL-1 and PDL-2, and potently suppressed T cell proliferation and IFN-γ secretion (39, 40). The regulatory phenotype of the APCs was due to iNKT cell release of extracellular ATP, which signaled through the P2X7 receptor on the monocytes to induce upregulation of PD-L1 and PD-L2 (41). This iNKT-monocyte interaction resembles a pathway observed in a murine model in which IL-13 secreted by CD1d-restricted T cells promoted monocyte expression of TGFβ, which led to suppression of T cell effector responses (42, 43), although the role of TGFβ in the human iNKT-monocyte pathway remains unclear.

We also used a xenotransplantation model of hematopoietic engraftment to investigate the impact of the human iNKT-monocyte pathway *in vivo*. The addition of allogeneic adult iNKT cells to human cord blood mononuclear cell grafts resulted in dramatically improved engraftment, which was due to iNKT cells inducing cord blood monocytes to secrete prostaglandin E_2_, which potently suppressed T cell IFN-γ production (44). Since hematopoietic engraftment is suppressed by excessive IFN-γ (45), this analysis shows that human iNKT cells can engage powerful regulatory pathways that limit adverse effects of human T_H1_ activation *in vivo*.

### iNKT cytolytic activity

A number of studies have suggested that iNKT cells may also control inflammation by eliminating pro-inflammatory APCs through a mechanism involving CD1d-dependent activation of the iNKT cells and lysis of APCs by cytotoxic granule deposition (46–50). Human iNKT cells were found to kill monocyte-derived DCs and blood DCs, but did not kill monocytes or plasmacytoid DCs, suggesting they specifically target certain types of APCs (46, 49). In another analysis, human iNKT cells preferentially eliminated monocyte-derived DCs that produced high levels of IL-12 while those that produced mainly IL-10 were spared, resulting in a DC population that limited T_H1_ activation (48). Together these studies suggest that this cytolytic pathway selectively targets pro-inflammatory APCs, and might thereby limit pathological inflammation. Consistent with this, in mice infected with a highly pathogenic strain of influenza A virus, iNKT cells were associated with reduced accumulation of inflammatory monocytes in the lungs (50). iNKT cell activity in this model was associated with reduced levels of MCP-1 (a chemokine that recruits monocytes and CD4^+^ T cells), reduced damage to lung tissue, and improved survival even though viral loads were not affected (50). The effect of iNKT cells was thought to be due to their cytolytic activity against influenza-infected monocytes, suggesting that iNKT cells may limit pathological inflammation during viral infections by eliminating inflammatory APCs. However, an important note is that in all of these studies the iNKT cells were experimentally exposed to strong TCR stimulation prior to analysis of their cytolytic activity. Therefore, the physiological conditions that might lead to APC-targeted cytolytic activity by iNKT cells remain unclear.

## How are iNKT cells activated, physiologically?

iNKT cells can be activated in two ways: either through TCR-mediated recognition of antigen presented by CD1d, or through TCR-independent pathways such as exposure to the cytokines IL-12 or IL-18, or LFA-1 ligation by high-density ICAM-1 (51–54). These TCR-independent pathways selectively induce iNKT cells to produce IFN-γ and not T_H2_ or regulatory cytokines (52, 54). Additionally, iNKT cells require a TCR signal for cytolysis of target cells (55–57). Thus, the anti-inflammatory activities of iNKT cells are probably highly dependent on TCR-recognition of antigens presented by CD1d molecules. Since it is clear that iNKT cells can mediate regulatory effects in the absence of infectious challenges, the antigens required for their anti-inflammatory pathways must be constitutively or chronically present. However, the sources and nature of the antigens that physiologically activate iNKT cells, and correspondingly the processes that contribute to their increased or decreased activation in different contexts, remain an ongoing area of inquiry.

### Sources of antigen

Due to their shared use of a canonical TCRα chain, all iNKT cells recognize an unusual type of glycolipid in which the sugar head group is present in an α-anomeric configuration. Certain microbes produce glycolipids of this type that are potent antigens for iNKT cells (reviewed in (8)). Recent studies indicate that bacterial species that can be found within the normal gut microbiota can produce similar antigenic lipids (58, 59), although these may be counter-regulated by related forms produced by other bacteria that are antagonists (60). These studies suggest that, particularly at mucosal sites, TCR-dependent activation of iNKT cells may fluctuate according to the composition of the microbial community.

iNKT cells can also recognize self-lipids as antigens. Mammalian cells do not directly synthesize the α-linked glycolipids recognized by iNKT cells, but the β-linked forms they produce may be converted at low frequencies to α-linked forms that are strongly antigenic (61, 62). Additionally, iNKT cells can recognize mammalian β-linked glycolipids as weak agonists (63). Some antigenic self-lipids, including lysophospholipids, glycosylated sphingolipids, and neutral lipids, are specifically upregulated during inflammation or cellular stress (64–70). Conversely, some non-antigenic self-lipids, such as sphingomyelin, can inhibit presentation of antigenic species (66). Together, the available data suggest that antigenic self-lipids are constitutively present, but are maintained in a manner that is only weakly agonistic for iNKT cells, and that during inflammation or cellular stress the abundance or nature of the antigenic self-lipids changes in a way that provides stronger TCR signals to iNKT cells. Additionally, as discussed below, activation by self antigens can be markedly enhanced by TCR-independent signals (71, 72).

## What determines the nature of the functional response mediated by iNKT cells?

Exposure to inflammatory cytokines (IL-12, IL-18) or elevated levels of the adhesion ligand ICAM-1 selectively promotes iNKT cell IFN-γ secretion (51, 52, 54, 71, 72). Thus, when these signals are present, such as during inflammation, iNKT cells probably predominantly promote inflammatory responses. In contrast, the TCR-dependent activation pathway can promote either pro-inflammatory or anti-inflammatory outcomes (reviewed in (73)), and it has been of considerable interest to understand how TCR-mediated activation of iNKT cells leads to these contrasting effects. Two central factors have emerged: first, that the iNKT cell population contains multiple functionally distinct subsets; and second, that iNKT cell functional responses vary according to antigen characteristics.

### Distinct subsets

**Figure 2:**
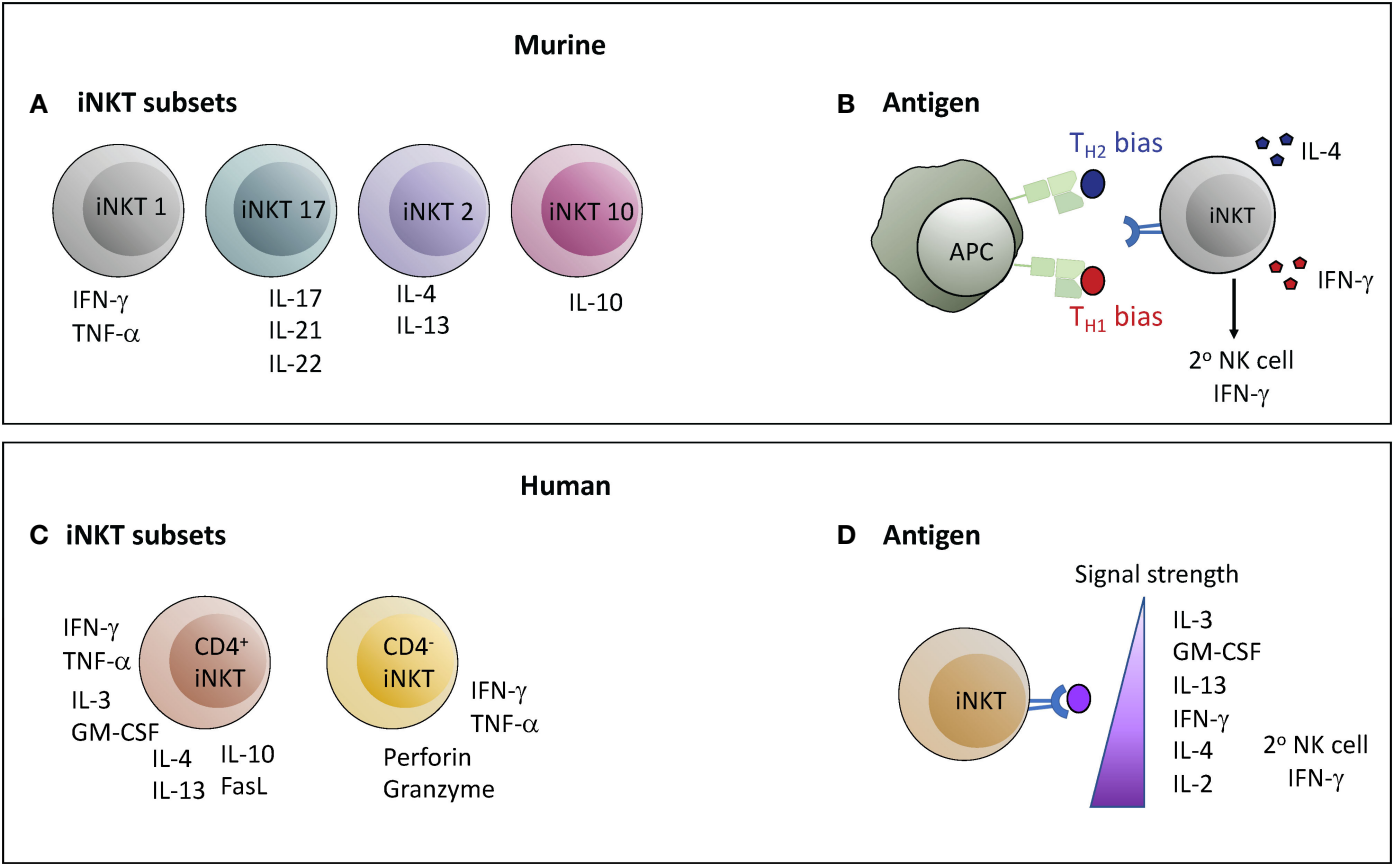
Determinants of the nature of the functional response mediated by iNKT cells. In both mice and humans the nature of the response mediated by iNKT cells may depend on the subset of iNKT cells activated or on the characteristics of the antigenic stimulation leading to activation. However, there are important differences between mice and humans in each of these parameters. **(A, C)** Murine iNKT cells can be classified into four lineages with functionally segregated cytokine profiles; whereas the two major subsets of human iNKT cells are characterized by comparatively polyfunctional cytokine production (CD4^+^) or a more T_H1_/cytotoxic profile (CD4^-^). **(B, D)** Structural features of lipid antigens can bias murine iNKT cell responses towards either a T_H1_ or T_H2_ output, whereas human iNKT cell cytokine production proceeds in a hierarchical manner depending on the strength of the TCR signal. Antigens that stimulate a T_H1_-biased response in mice typically also produce a strong secondary wave of IFN-γ production by NK cells, whereas strong agonists produce this effect from human iNKT cells.

In contrast to conventional T cells that become polarized into different effector phenotypes by priming in the periphery, iNKT cells are already cytokine competent as they exit the thymus (74). Murine iNKT cells are segregated into four functionally distinct subsets based on their expression of master-regulator transcription factors that govern cytokine production (Tbet, GATA3, RORγT, E4BP4) and on differences in expression levels of PLZF (promyelocytic leukemia zinc finger), a transcription factor that promotes cellular characteristics associated with innate lymphocytes (28, 75–78). NKT1 cells have a T_H1_ cytokine profile, often express a cytotoxic effector program, and are PLZF^lo^Tbet^hi^; NKT2 cells are characterized by high levels of IL-4 secretion and are PLZF^hi^GATA3^+^; NKT17 cells produce IL-17 and express RORγT with intermediate levels of PLZF; NKT10 cells produce IL-10, are preferentially found within adipose tissues, and are negative for PLZF but express E4BP4 (**Figure 2A**). NKT1, NKT2, and NKT17 lineages are generated during thymic selection, and are thought to home to distinct tissues (79). In contrast, NKT10 cells may originate from other subsets and differentiate into a regulatory phenotype as a result of exposure to factors in adipose tissues (27). The identification of these iNKT sub-lineages has led to the paradigm that the anti-inflammatory effects of iNKT cells are due to NKT2 or NKT10 cells, which become activated in different situations than NKT1 and NKT17 subsets as a result of differences in tissue localization.

In contrast, it has thus far not been straightforward to categorize human iNKT cells into NKT1, NKT2, and NKT17 lineages matching those in mice. Similar to their murine counterparts, most human iNKT cells express PLZF (80–82), and are characterized by an innate-like transcriptional profile that results in a “poised-effector” status allowing them to rapidly mediate functional responses (83). Multi-parameter flow cytometric analyses and gene expression studies have revealed human iNKT cells to express a diverse selection of cytokines and chemokines (84–88). Human iNKT cells can be segregated into two major subsets according to CD4 expression (84, 85). Those that express CD4 often appear to co-produce GM-CSF, IL-13, TNF-α, IFN-γ, IL-4, and IL-2, while those lacking CD4 appear more specialized for cytolysis (**Figure 2C**). These two major populations are sub-divided into further subsets characterized by additional markers (e.g. CD8α, CD161, CD62L) with distinctions in functional characteristics, but it is not clear that these subsets equate to the NKT1, NKT2, or NKT17 lineages observed in mice (89, 90). It is also not clear whether anti-inflammatory activity segregates according to CD4^+^ or CD4^-^ status of human iNKT cells, although CD4^+^ iNKT cells are the ones that have been found to induce regulatory functions in monocytic cells, and the CD4^-^ subset has appeared more likely to kill DCs.

### Antigenic modulation

The prototypical iNKT antigen is called α-galactosylceramide (α-GalCer) (91), and synthetic forms of this lipid have proved extremely valuable as pharmacological agents that activate iNKT cells in a highly specific manner (92). Observations that structural variants of α-GalCer can produce substantially different immunological outcomes *in vivo* have led to interest in using these agents to selectively tune iNKT responses towards pro- or anti-inflammatory functions (92). Administration of α-GalCer to mice potently stimulates iNKT cells, and induces a mixed response where T_H1_, T_H2_, and regulatory cytokines are all produced, although with different kinetics (93). In contrast, certain analogues of α-GalCer have been shown to produce a T_H2_-biased cytokine response (94, 95), while other variants produce a highly T_H1_-biased response (96) (**Figure 2B**). The mechanisms underlying these differential responses appear complex. One component may be that certain variants induce biased cytokine production from iNKT cells themselves (97), while another important element likely relates to whether or not antigen-driven interactions between iNKT cells and APCs result in release of cytokines (e.g. IL-12) that activate a secondary IFN-γ response by NK cells (96, 98). A key factor may be the relative duration of antigen presentation by CD1d molecules, with more durable antigens being associated with T_H1_-biased responses (99). Additionally, T_H1_-biasing forms of α-GalCer may be selectively presented by APCs that produce IL-12, whereas T_H2_-biasing forms may be more promiscuously presented and thus less likely to produce a secondary wave of IFN-γ production by NK cells (100). It is not clear whether antigen variants selectively activate different iNKT cell subsets, or bias the cytokine profile produced within a given subset (for example, by inducing higher IL-4 production by NKT1 cells, or increased IFN-γ by NKT2 or NKT17 cells), or whether any structural variants selectively promote IL-10 production. Interestingly, repeated administration of α-GalCer results in selective loss of its T_H1_-promoting features, but under such “anergizing” conditions α-GalCer retains the ability to induce IL-4 secretion and to promote control of EAE pathology (101).

Whether human iNKT cell responses can be modulated similarly using α-GalCer structural variants remains an open question. It has become clear that TCR differences between murine and human iNKT cells result in significant discrepancies in TCR-signaling strength induced by lipid variants (102). Perhaps more importantly, polyfunctional human iNKT cells show a hierarchy of cytokine production in response to TCR stimulation that does not neatly segregate into clear T_H1_ or T_H2_ patterns. Weak TCR stimulation of human iNKT cells preferentially induces production of IL-3, GM-CSF, and IL-13, with increasing stimulation leading first to IFN-γ, then IL-4, then IL-2 (44, 103, 104) (**Figure 2D**). Secondary induction of NK cell IFN-γ secretion was associated with activation of human iNKT cells by strong TCR agonists (104). It is therefore not clear that it will be feasible to selectively polarize human immune responses towards IL-4 production through the use of specific lipid antigen variants, although it may be possible to drive IL-13 production through administration of weak agonists.

## Discussion

The potential of engaging iNKT cells therapeutically to treat T_H1_-inflammatory pathology is well supported by pre-clinical studies in murine models, *in vitro* experiments using human cells, and *ex vivo* analyses of human subjects, but clinical data have been limited. Recently, however, a pilot clinical trial using allogeneic iNKT cells as a cellular immunotherapy to treat patients who were intubated with acute respiratory distress syndrome (ARDS) secondary to SARS-CoV-2 infection has shown highly promising results, with 77% survival of treated patients compared to a national average of 40% survival for other intubated SARS-CoV-2 patients during the same period of enrollment (105). Understanding whether such iNKT cell therapies work through one of the regulatory pathways shown in **Figure 1A**, or through elimination of inflammatory cells *via* cytolysis as depicted in **Figure 1B**, has important implications. For example, if APC killing is a key component it may be necessary to deliver a strong TCR signal to the iNKT cells to prime their cytolytic activity. Alternatively, if a regulatory pathway is involved it may be beneficial to generate iNKT cells that are biased towards production of T_H2_ cytokines or IL-10, depending on the pathway.

Also critical to developing effective iNKT cell therapies is to determine whether human iNKT cells include stable regulatory subsets, or whether polyfunctional iNKT cells are converted into a regulatory phenotype through particular signals. If a stable NKT10 lineage exists in humans, an attractive option might be to specifically engage these cells for immunotherapy. Alternatively, if human iNKT cells generally retain functional plasticity, it may be important to identify methods to specifically promote their regulatory functions. To this end, a recent study found that the presence of IL-7 during *in vitro* expansion of human iNKT cells resulted in a CD4^+^ population with enhanced T_H2_ cytokine production (106), while exposure to short chain fatty acids, palmitate, or the mTOR inhibitor rapamycin may induce a regulatory phenotype (27, 37, 107). Another important consideration is that iNKT immunotherapy that engages T_H2_ pathways would likely be contraindicated in certain inflammatory diseases, including asthma, chronic obstructive pulmonary disease, and ulcerative colitis, where T_H2_ cytokine production by iNKT cells has been associated with disease-exacerbating effects (reviewed in (108–110)).

Overall, studies of human and murine iNKT cells over the last three decades clearly support the potential of this unique population to be utilized clinically to control inflammatory pathology. Key areas of further investigation will be to better understand the antigens that physiologically or pharmacologically activate human iNKT cells, and to determine the impact of iNKT cell antigenic activation in different tissues or by distinct APCs. For example, since lipid antigens can be retained locally at the site of administration (111), or distributed to distal sites through binding to lipid transport proteins (112, 113), it may be possible to control the location of iNKT cell activation. Additionally, since iNKT cells promote anti-inflammatory outcomes through interactions with multiple distinct APC populations, it may be possible to direct specific effects through engaging particular APC types, such as the regulatory B cells that ameliorate arthritic pathology (114). It will also be of importance to understand roles of non-invariant populations of CD1d-restricted T cells (often called type II NKT cells, reviewed in reference (115)), and to determine whether these other T cell populations promote or counter-regulate anti-inflammatory outcomes mediated by the “type I” iNKT cells discussed here. Finally, given the likely importance of TCR and CD1d structural differences, the difference in abundance between murine and human iNKT cells (common experimental mouse strains have ~100-fold higher frequencies of iNKT cells than humans), and of additional CD1 molecules (CD1a,b, c, and e) expressed in humans that may impact antigen availability or T cell responses (7, 116, 117), an important step for translating iNKT-based immunotherapies to the clinic may be the development of new animal models that better capture determinants that affect human iNKT cell functions.

## Author contributions

NSB and JEG wrote the manuscript and generated the figures. All authors listed have made a substantial, direct, and intellectual contribution to the work and approved it for publication.

## Funding

Major support provided by NIH R01 AI136500 to JG; NB also supported by funding from the University of Wisconsin-Madison’s Office of the Vice Chancellor for Research and Graduate Education through the Fall Competition Program.

## Conflict of interest

Author JG is a member of the Scientific Advisory Board of MiNK Therapeutics.

The remaining author declares that the research was conducted in the absence of any commercial or financial relationships that could be construed as a potential conflict of interest.

## Publisher’s note

All claims expressed in this article are solely those of the authors and do not necessarily represent those of their affiliated organizations, or those of the publisher, the editors and the reviewers. Any product that may be evaluated in this article, or claim that may be made by its manufacturer, is not guaranteed or endorsed by the publisher.
